# The agonizing effects of uncertainty: Effects of announced vs. unannounced performance assessments on emotions and achievement

**DOI:** 10.1371/journal.pone.0272443

**Published:** 2022-08-17

**Authors:** Maik Bieleke, Jean-Marie Schwarzkopf, Thomas Götz, Ludwig Haag

**Affiliations:** 1 Department of Sport Science, University of Konstanz, Konstanz, Germany; 2 Gymnasium Eppendorf, Hamburg, Germany; 3 Department of Developmental and Educational Psychology, Faculty of Psychology, University of Vienna, Vienna, Austria; 4 Department of Educational Sciences, Faculty of Humanities and Social Sciences, University of Bayreuth, Bayreuth, Germany; UNITED STATES

## Abstract

Performance assessments play an essential role in performance diagnostics at schools. In practice, both announced and unannounced assessments are regularly used. However, it is unclear whether assessments are better administered announced or unannounced. From a theoretical perspective, it can be argued that announced assessments, mediated by the greater degree of students’ subjective control that accompanies them, should have a more beneficial effect on emotions, as well as the subsequently resulting performance, than unannounced assessments. To investigate these assumptions, emotion (enjoyment, anxiety) and achievement data (grades) were collected from 414 students in 19 lower- and upper-level tracks at a German secondary school on both announced and unannounced performance assessments. Less anxiety and more enjoyment occurred on the announced assessments. Moreover, enjoyment and anxiety were predictors of performance (i.e., school grades), which was better overall on announced assessments than on unannounced ones. The results of our field study suggest that announced assessments have more beneficial effects on emotions than unannounced assessments.

## Introduction

Questions regarding effective teaching and teacher training have been booming in re-search for at least 20 years [1]. However, little consideration has been given to issues of performance diagnostics in this regard, although its practical importance is extremely high [2]. In purely quantitative terms, performance assessment at schools also takes up a large amount of resources—after all, the number of assessments that German students have to take in their nine-year high school career up to the Abitur is probably around 1,000. In performance diagnostics, the question of adequate performance assessment and its implications has a now century-old history [3]. In the German research tradition, for example, empirical research that focused on the questionability of grading became well-known [4]. In contrast, the issue of performance assessment has received relatively little attention. Here, the focus has been mainly on improving grading in terms of transparency and matching demands with learning effort [5]. Apparently, there is no study on the question to what extent performance assessments should be announced—at least, no studies on this can be found in common research databases. However, this issue is highly relevant from a scientific as well as a practical perspective. From a theoretical perspective, the announcement vs. non-announcement of performance assessments should have effects on students’ emotions (e.g., enjoyment, anxiety) and their resulting performance outcomes based.

The question of whether performance assessments should be announced or held unannounced, which affects everyday teaching, is not a trivial one. This is probably illustrated best by the different ways in which assessments are handled in the different federal states in Germany. In general, schools distinguish between written, oral, and practical assessments. The written assessments, which are also vaguely called tests, are divided into major and minor performance assessments. The first group includes class and school assignments or exams—the terms vary depending on the federal states. The second group includes short assessments (impromptu tasks). Unannounced performance assessments are not permitted in all federal states. While in Hamburg, for example, all written assessments must be announced and unannounced performance assessments are not permitted, in Bavaria, impromptu assessments can be held without announcement.

There are arguments for both approaches: The argument in favor of an unannounced procedure is that the students should learn constantly and that a written assessment on the subject matter of the last lessons should not be a problem. The main argument against unannounced assessments is the presumed constant feeling of insecurity and fear—such a constant "beware" attitude is not likely to provide much joy for students. In the case of unannounced examinations, it can therefore be assumed that negative emotions predominate.

Emotions play an important role in teaching and generally in learning and performance situations (e.g., [6, 7]). Anxiety and especially test anxiety have been studied by far the most within research on academic emotions ([8]; cf. the tradition of test anxiety research since the 1930s [9]; and then increasingly since the 1950s [10]). There are also relatively many studies on enjoyment, compared to other emotions. According to Pekrun’s [11, 12] control-value theory, academic emotions show effects on self-regulated learning, including the use of learning strategies, as well as on motivation (e.g., intrinsic vs. extrinsic motivation), and the availability of cognitive resources needed for working on tasks. Specific emotions require different amounts of cognitive resources. Test anxiety, for example, requires a great amount of resources through the worries that accompanies it (worry-cognitions; [13, 14])–in fact, worries have been shown to be a defining characteristic of test anxiety (e.g., [15]). In sum, emotions can strongly be assumed to influence performance via the mentioned processes.

Meta-analyses show that anxiety is in fact detrimental for performance (e.g., [16, 17]). In line with the mechanisms as mentioned above, anxiety requires many cognitive resources, reduces intrinsic motivation, and leads to monotonous, rigid (i.e., very detail-focused) learning behavior. However, it is repeatedly argued that anxiety can also be beneficial for performance by increasing extrinsic motivation in specific situations [12]. The net effects of anxiety on performance depend on which of the above mediating mechanisms are predominate. As mentioned, meta-analyses clearly show that mechanisms leading to negative effects on performance usually prevail. Enjoyment, on the other hand, is clearly positively related to performance outcomes. It requires relatively few cognitive resources, promotes intrinsic motivation, and leads to flexible learning behaviors, such as high levels of self-regulation and the use of elaboration strategies [18].

Central to the emergence of anxiety and enjoyment, as well as other emotions, is the subjective control that students experience. High levels of subjective control increase positive and reduce negative emotions. Vice versa, a low level of subjective control increases negative and reduces positive emotions. Control here means the extent to which one can predict and personally influence events [12, 19, 20]. For instance, students become increasingly anxious and worried about their grades after an examination, that is, when they have no longer have control over the outcome (a phenomenon known as the incubation of threat [21]). This link between control and emotions is emphasized in at least four different research traditions: research on locus of control [22–24], research on self-efficacy beliefs [19, 25, 26], research on attribution theories [27], and research on appraisal-based emotion theories (e.g., [11, 12, 28]). In schools, subjective control can be increased or decreased in multiple ways. Arguably, announced vs. unannounced performance assessments are an important aspect of students’ subjective experience of control in school.

As compared to unannounced performance assessments, announced performance assessments are objectively and subjectively associated with a much higher degree of control. Students know when they are going to take place and can prepare accordingly, which subjectively gives them more control over the exam itself. As a result of this high level of control, high levels of enjoyment and low levels of anxiety can be expected. Both emotions should in turn show corresponding effects on the performance results: While enjoyment should lead to better performance, anxiety should rather reduce it. But also beyond the emotions, the announcement of tests should result in higher control and lead to better performance with regard to the possibility of targeted preparation for the test.

## Hypotheses

Based on the theoretical assumptions as outlined above, the following hypotheses can be made: There should be less anxiety in announced performance assessments as compared to unannounced performance assessments (Hypothesis 1). More enjoyment should be experienced in announced performance assessments as compared to unannounced performance assessments (Hypothesis 2). According to the assumed effect of emotions on performance, higher anxiety should reduce performance and higher enjoyment should increase performance (Hypothesis 3).

## Methods

Our study was conducted in accordance with the principles of Declaration of Helsinki and the American Psychological Association (APA) and was approved by the ethics committee at the University of Konstanz (IRB21KN12-005w). Written parental consent was obtained prior to the study and students provided verbal informed consent.

### Participants

Data were collected from 414 students (53.4% female, 46.6% male) from 11 lower- (Grade 8, 9, and 10) and 8 upper-level tracks (Grade 11) at a Gymnasium in Hamburg, Germany. The Gymnasium is the high-achieving track of the three-track German secondary school system. Approximately 40% of the total student cohort attend this track [29]. For data protection reasons (ensuring anonymity), age was not assessed. Data collection took place by the teacher during regular class time. Written parental consent was a prerequisite for voluntary participation in the study; we received consent from 414 of 417 students (99.3%). Data were collected in the three school subjects of economics, geography and politics, society and business.

### Instruments

Enjoyment was assessed with three items (e.g., "I enjoy some of the topics in this subject so much that I look forward to them" [30]) and anxiety was recorded with six items (e.g., "I would prefer not to go to school because of my fear of this subject" [31, 32]). The corresponding statements were rated on a four-point Likert scale ranging from 1 (*strongly disagree*) to, 4 (*strongly agree*). Cronbach’s alpha was acceptable across all time points for both enjoyment (α = .74 to .83) and anxiety (α = .75 to .84).

Academic performance was assessed using students’ grades on the unannounced and announced performance assessments. Grades range from 1, very good, to 6, insufficient, with higher numbers representing poorer performance. To ease interpretation, we inverted grade scores so that higher numbers indicated better performance. Performance assessed in this study is both valid and of practical interest in that it corresponds to the common practice of teachers who design written assessments on their own.

### Procedure

The data collection included four measurement time points per person. Two weeks prior to the unannounced performance assessment (Time Point 1), students were informed that an unannounced performance assessment would take place in class at any time during the first half of the school year. Students then completed the first questionnaire. After the two weeks had passed, that is, on the day of the unannounced performance assessment (Time Point 2), the teacher told the class at the beginning of the lesson that a performance assessment was now about to take place. Before taking this test, the students again completed the questionnaire they had already been given two weeks ago. The duration of the subsequent performance assessment was about 30 minutes.

At the beginning of the second half of the school year, these same students were informed that there would now no longer be an unannounced written performance assessment in class. Rather, they would be notified two weeks in advance that there would be a performance assessment. Accordingly, the assessment was announced to the students two weeks before the performance assessment and they completed the third questionnaire on the day of this announcement (Time Point 3). On the day of the performance survey, they filled out the fourth questionnaire right before taking the test (Time Point 4). The performance survey lasted about 45–60 minutes in the lower-level tracks and about 90 minutes in the upper-level tracks. Performance assessments and questionnaires were handed out as paper-pencil versions. All performance assessments were graded by the subject teaches.

### Data analysis

Data were processed and analyzed in the statistical software environment R (version 4.0.4 [33]). The effects of the type of performance assessment (announced vs. unannounced) and the time point (two weeks before the assessment vs. on the day of the assessment) on the two emotions of enjoyment and anxiety were examined using mixed linear regression models. Mixed linear regression models were also used for the analysis of academic performance, with enjoyment and anxiety (two weeks before the assessment vs. on the day of the assessment) and the type of performance assessment (announced vs. unannounced) as predictors. All significance tests were two-sided, and α was set to .05.

## Results

Descriptive statistics (means, standard deviations, and correlations) of all variables are presented in Table 1. Moderate to large correlations of the four time points were found with respect to enjoyment (r = .70 to .83) as well as with respect to anxiety (r = .44 to .67). The correlations between enjoyment and anxiety are consistently negative and range from low to medium (r = -.01 to -.31). Academic achievement correlated positively and strongly with enjoyment (r = .51 to .65) and negatively and weakly with anxiety (r = -.02 to -.14). With the present sample size (N = 414), correlations are statistically significant at a value of r ≥ |.096|.

**Table 1:** Descriptive statistics of main variables.

Variable	*M*	*SD*	1	2	3	4	5	6	7	8	9	10
1. Enjoyment (1)	3.06	0.57										
2. Enjoyment (2)	2.59	0.63	**.74**									
			[.69,.78]									
3. Enjoyment (3)	3.05	0.60	**.79**	**.72**								
			[.75,.82]	[.67,.76]								
4. Enjoyment (4)	2.85	0.60	**.70**	**.70**	**.83**							
			[.65,.75]	[.65,.75]	[.80,.86]							
5. Anxiety (1)	2.08	0.57	-.08	**-.27**	-.01	-.03						
			[-.18,.02]	[-.36,-.18]	[-.11,.09]	[-.13,.07]						
6. Anxiety (2)	2.57	0.66	-.04	**-.31**	-.01	-.04	**.67**					
			[-.14,.06]	[-.39,-.22]	[-.11,.09]	[-.14,.06]	[.61,.72]					
7. Anxiety (3)	1.56	0.44	**-.18**	**-.16**	**-.20**	**-.14**	**.48**	**.44**				
			[-.27,-.08]	[-.25,-.06]	[-.29,-.11]	[-.23,-.04]	[.41,.56]	[.36,.52]				
8. Anxiety (4)	1.91	0.53	**-.11**	**-.17**	-.08	-.06	**.54**	**.56**	**.64**			
			[-.20,-.01]	[-.27,-.08]	[-.17,.02]	[-.16,.04]	[.47,.61]	[.49,.63]	[.58,.70]			
9. Academic Performance (2)	3.06	1.01	**.58**	**.56**	**.65**	**.55**	-.02	**-.13**	**-.13**	**-.13**		
			[.51,.64]	[.49,.63]	[.58,.70]	[.48,.62]	[-.12,.08]	[-.23,-.03]	[-.22,-.03]	[-.23,-.03]		
10. Academic Performance (4)	2.46	0.99	**.58**	**.59**	**.62**	**.51**	-.04	-.05	-.08	**-.14**	**.79**	
			[.51,.64]	[.52,.65]	[.55,.67]	[.44,.58]	[-.14,.06]	[-.15,.04]	[-.18,.01]	[-.24,-.05]	[.75,.82]	
11. Gender	1.53	0.50	-.02	-.02	-.03	-.03	**.10**	.03	.02	.01	.01	-.03
			[-.12,.08]	[-.12,.07]	[-.13,.07]	[-.13,.06]	[.01,.20]	[-.07,.13]	[-.08,.12]	[-.09,.11]	[-.09,.11]	[-.12,.07]

*Note*. (1) = two weeks before the unannounced performance assessment; (2) = on the day of the unannounced performance assessment; (3) = two weeks before the announced performance assessment; (4) = on the day of the announced performance assessment. Bold correlations are significant, 95% confidence intervals are provided in square brackets. Answers were given on the following scales: Enjoyment and anxiety: from 1 = *strongly disagree* to 4 = *strongly agree*. Academic performance: inverted grades, such that higher values correspond to better performance; non-inverted scale: 1 = *very good* to 6 = *insufficient*. Gender: 1 = male, 2 = female.

### Achievement emotions

**Fig 1:**
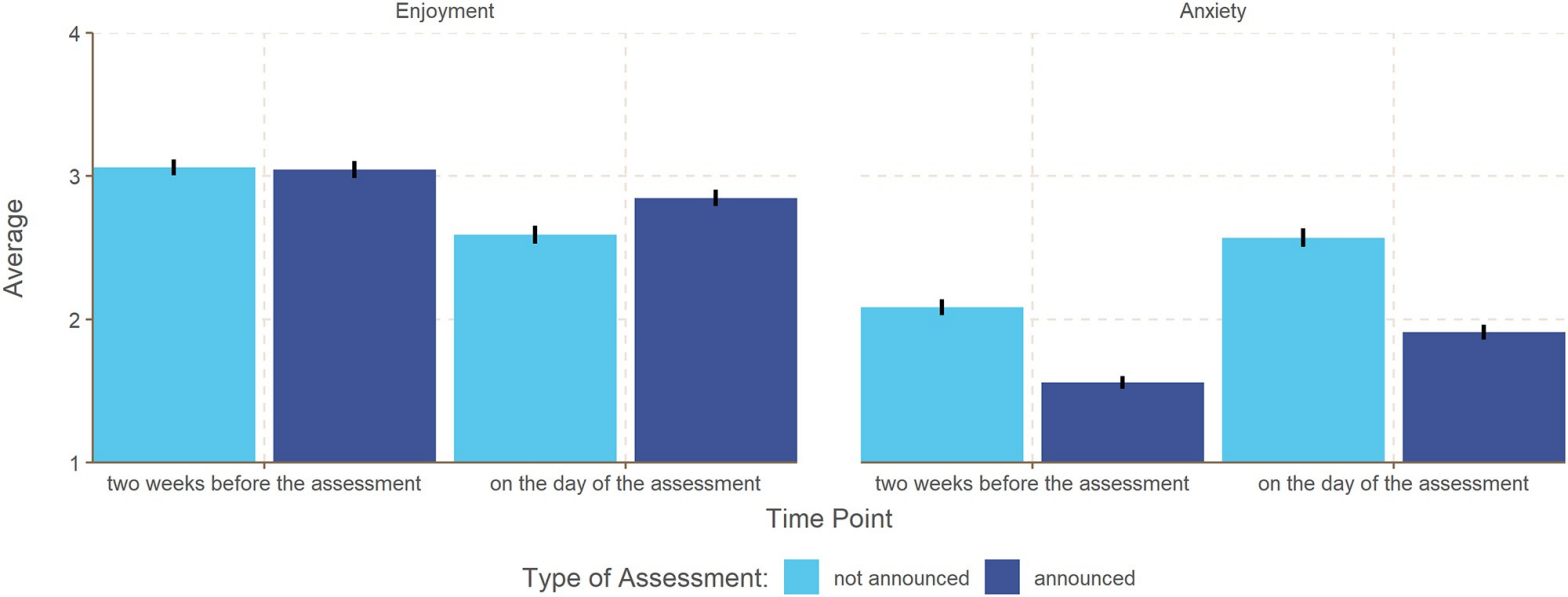
Average enjoyment and anxiety as a function of the type of assessment and the time point. Error bars represent 95% confidence intervals. Response scales for enjoyment and anxiety: 1 = *strongly disagree* to 4 = *strongly agree*.

The analysis of enjoyment showed a significant interaction between the type of assessment and the time point, *b* = 0.282, *SE* = 0.031, *p* < .001 (see also Fig 1). This interaction is due to the fact that there were no differences between announced and unannounced performance assessments with regard to enjoyment two weeks before the assessment, *b* = -0.023, *SE* = 0.022, *p* = .295, whereas enjoyment on the day of the assessment was higher for an announced performance assessment than for an unannounced assessment, *b* = 0.259, *SE* = 0.022, *p* = < .001.

The analysis of anxiety also showed a significant interaction between the type of assessment and the time point, *b* = -0.132, *SE* = 0.037, *p* < .001. However, anxiety was lower both two weeks before the performance assessment, *b* = -0.531, *SE* = 0.026, *p* < .001, and on the day of the performance assessment, *b* = -0.663, *SE* = 0.026, *p* < .001, for an announced assessment compared to an unannounced assessment. Results for both enjoyment and anxiety did not change when controlling for gender.

### Academic performance

**Fig 2:**
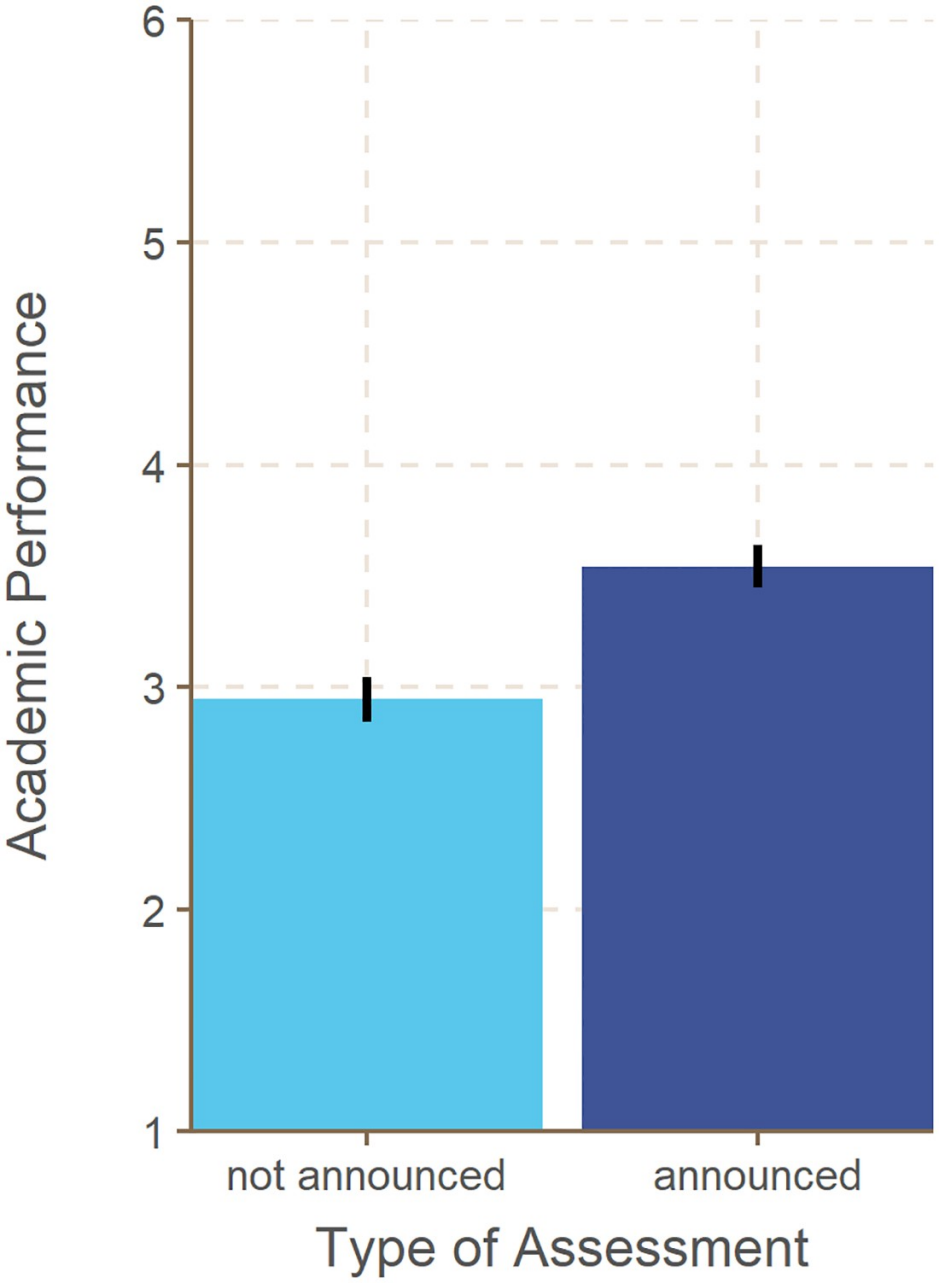
Academic performance as a function of the type of assessment. Error bars represent 95% confidence intervals. Academic performance reflects inverted grades such that higher values correspond to better performance.

Better performance was predicted by higher levels of enjoyment two weeks before the performance assessment, *b* = 0.630, *SE* = 0.070, *p* < .001, and also by higher levels of enjoyment on the day of the performance assessment, *b* = 0.225, *SE* = 0.066, *p* < .001. Higher levels of anxiety two weeks before the performance assessment also predicted better performance, *b* = 0.318, *SE* = 0.063, *p* < .001, whereas higher levels of anxiety experienced on the day of the performance assessment predicted poorer performance, *b* = -0.285, *SE* = 0.056, *p* < .001. For these results, the type of assessment was controlled for. Announced assessments were associated with better scores than unannounced assessments, *b* = 0.534, *SE* = 0.050, *p* < .001 (Fig 2).

## Discussion

The question of performance diagnostics has received little research attention in recent years, despite its great practical relevance [2]. Here, we focused on the question of how announced vs. unannounced performance assessments affect emotions and grades. Based on theoretical assumptions about the link between control, emotion, and performance (e.g., in the control value theory of achievement emotions; [11, 12]), three hypotheses were formulated for this purpose. With this study, the hypotheses can be confirmed that there is less anxiety and more enjoyment in announced as compared to unannounced performance assessments. Further, anxiety reduces performance and enjoyment increases performance. Thus, the results of this study are consistent with the decrees of the German ministries of education that unannounced written performance surveys are not permitted. Performance assessments strategies based on Matthew 25:13 ("Be watchful, for you know neither the day nor the hour"), therefore, seem not advisable.

Only anxiety experienced two weeks prior to the unannounced performance assessment had a positive effect on performance. This result points to the ambivalent effects of anxiety on performance as outlined in the introduction [12]. With regard to preparation for performance assessments, it may increase extrinsic motivation and thus the amount of preparation, which may well result in higher performance. In the actual assessment situation, however, anxiety reduces performance, primarily because it consumes cognitive resources (e.g., worry-cognitions; [13]). It is also noteworthy that performance was more strongly associated with enjoyment than with anxiety, which might be counterintuitive at first glance. However, this observation likely reflects that the association between performance and enjoyment is straightforward (higher levels of enjoyment are unambiguously linked to better performance), while the association between performance and anxiety is ambivalent and therefore weaker: While anxiety can facilitate performance by providing extrinsic motivation, it might also hamper performance by inducing worry and fear. This ambivalence should result in a comparatively weak link between performance and anxiety (e.g., [17]).

For the school to fulfill its function of selection, unannounced performance assessments are not necessary. Selection can occur in clearly timed and communicated tests. And as alternative ways of assessing performance developed in the context of a new learning culture become further established, the problem will become completely superfluous eventually: Assessment formats such as learning reports, portfolio work, discussions, cooperatively performed tasks, and student self-assessments provide for a different temporal framing than a performance assessment occurring at a fixed point in time. However, one argument against the plea for announced performance assessments is worth mentioning. Traditional final assessments naturally tempt students to memorize facts and knowledge in the short term, which are then recalled during the assessment. This follows Seneca’s phrase "Non vitae, sed scholae discimus ("Not for life, but for school do we learn;" [34]). It would be worthwhile to study the impact on emotions and performance of a classroom in which learning occurs but which refrains from scheduled assessments in favor of process-oriented assessments.

Some limitations of the present study should be noted. They might be helpful with respect to potential avenues for future research in this field. First, standardized assessments might be used instead of grades in future studies to hedge against, for instance, possible differences in how lenient announced versus unannounced tests are graded by teachers. Second, subjective control, which was not assessed in this study, could be integrated as a variable to investigate the presumed antecedents of enjoyment and anxiety. Moreover, it is plausible that positive effects of announcing an examination on performance depend on the degree to which students feel in control (e.g., the possibility to get help from peers). Third, we focused on measuring emotions and performance but did not collect data about other mechanisms that might help to explain our results. For instance, the control-value theory of achievement emotions [11, 12] assumes that motivational processes (e.g., effort invested in studying) mediate the effects of emotions on performance. Moreover, it is possible that differences in performance reflect differences in how much time students devoted to preparing for announced versus unannounced assessments. In future research, it would be worthwhile to assess potential confounding and mediating variables. Fourth, it should be noted that the observed differences between announced and unannounced performance assessments may also reflect other differences between the two assessments. For instance, the unannounced assessment always took place in the first semester, while the announced assessment took place in the second semester. Better performance in the announced assessment might thus reflect that students became more familiar with how their teacher designed problems and graded tests. An experimental study might solve this problem by randomizing announced versus unannounced tests (see [35], for such an approach). Fifth, although the associations between achievement emotions and their antecedents (e.g., differences in the kind of assessment) and consequences (e.g., performance) tend to be stable across subjects (the principle of structural equivalence [11]; for empirical support see [36]), it still seems worthwhile to replicate our findings in core subjects like mathematics and language classes. Similarly, larger studies that can account for student grouping in classes (e.g., using multilevel analyses) would be desirable to examine the robustness of our findings.

## Conclusion

Overall, the results of the present study present important initial evidence that unannounced compared to announced performance assessments may have negative effects on emotions (i.e., less enjoyment, more anxiety) and performance (i.e., grades). This evidence can be used by educational decision makers and teachers who have to balance conflicting goals like facilitating a continuous learning process (e.g., by administering unannounced tests) versus enhancing the emotional well-being of students (e.g., by announcing tests). For researchers, the present study provides a call to focus more strongly on the emotional aspects on performance assessment, which have been largely neglected in the literature.
